# Multi-omic validation of the cuproptosis-sphingolipid metabolism network: modulating the immune landscape in osteosarcoma

**DOI:** 10.3389/fimmu.2024.1424806

**Published:** 2024-06-25

**Authors:** Qingbiao Li, Jiarui Fang, Kai Liu, Peng Luo, Xiuzhuo Wang

**Affiliations:** ^1^ Department of Orthopedics, Southern Medical University Pingshan Hospital (Pingshan District Peoples’ Hospital of Shenzhen), Shenzhen, Guangdong, China; ^2^ Department of Sport Medicine, Huazhong University of Science and Technology Union Shenzhen Hospital (Nanshan Hospital), Shenzhen, China

**Keywords:** cuproptosis, sphingolipid, multi-omics, immune, metabolism, osteosarcoma

## Abstract

**Background:**

The current understanding of the mechanisms by which metal ion metabolism promotes the progression and drug resistance of osteosarcoma remains incomplete. This study aims to elucidate the key roles and mechanisms of genes involved in cuproptosis-related sphingolipid metabolism (cuproptosis-SPGs) in regulating the immune landscape, tumor metastasis, and drug resistance in osteosarcoma cells.

**Methods:**

This study employed multi-omics approaches to assess the impact of cuproptosis-SPGs on the prognosis of osteosarcoma patients. Lasso regression analysis was utilized to construct a prognostic model, while multivariate regression analysis was applied to identify key core genes and generate risk coefficients for these genes, thereby calculating a risk score for each osteosarcoma patient. Patients were then stratified into high-risk and low-risk groups based on their risk scores. The ESTIMATE and CIBERSORT algorithms were used to analyze the level of immune cell infiltration within these risk groups to construct the immune landscape. Single-cell analysis was conducted to provide a more precise depiction of the expression patterns of cuproptosis-SPGs among immune cell subtypes. Finally, experiments on osteosarcoma cells were performed to validate the role of the cuproptosis-sphingolipid signaling network in regulating cell migration and apoptosis.

**Results:**

In this study, seven cuproptosis-SPGs were identified and used to construct a prognostic model for osteosarcoma patients. In addition to predicting survival, the model also demonstrated reliability in forecasting the response to chemotherapy drugs. The results showed that a high cuproptosis-sphingolipid metabolism score was closely associated with reduced CD8 T cell infiltration and indicated poor prognosis in osteosarcoma patients. Cellular functional assays revealed that cuproptosis-SPGs regulated the LC3B/ERK signaling pathway, thereby triggering cell death and impairing migration capabilities in osteosarcoma cells.

**Conclusion:**

The impact of cuproptosis-related sphingolipid metabolism on the survival and migration of osteosarcoma cells, as well as on CD8 T cell infiltration, highlights the potential of targeting copper ion metabolism as a promising strategy for osteosarcoma patients.

## 1 Introduction

Osteosarcoma, an intensely aggressive malignant tumor originating from bone, predominantly affects adolescents (1–3). Recent studies have revealed that disruptions in metabolic pathways and alterations in the immune microenvironment significantly contribute to the pathogenesis of osteosarcoma (4–7). In the tumor microenvironment, tumor cells reprogram both the immune and metabolic landscapes to augment their proliferative, survival, and metastatic potential (8–10).

Sphingolipids are critical components of the cellular membrane surface, essential for maintaining the structural integrity of sphingolipids (11, 12). Sphingolipids and their metabolic derivatives are involved in regulating processes including apoptosis and autophagy. Sphingolipids and their metabolic derivatives are involved in regulating processes including apoptosis and autophagy (13, 14). The metabolism of sphingolipids also plays a role in the interactions between tumor cells and the immune system, influencing tumor cell behavior and the functionality of immune responses (15–17). Regulation of these metabolic pathways may reveal new therapeutic strategies for osteosarcoma.

Copper is an essential trace element involved in the activity and function of multiple enzymes, significantly impacting cellular energy production, neural conduction, antioxidative defense, and the absorption and metabolism of iron (18–21). The relationship between copper ion metabolism and tumor development has become increasingly clear, particularly with the discovery of a new mode of cell death induced by copper-cuproptosis (22, 23). Cuproptosis involves copper directly binding to certain intracellular fatty acid synthases, leading to protein aggregation and cellular dysfunction (24). This mechanism may provide targets for developing new therapies against refractory tumors such as osteosarcoma.

Studies indicate that metal ions like copper and iron can affect sphingolipid metabolism. These metal ions are involved in regulating the activity of enzymes directly related to sphingolipid metabolic pathways, affecting cell signaling and the physical properties of membranes. Additionally, copper ions are hypothesized to exert an indirect influence on the synthesis and degradation pathways of sphingolipids. Recent discoveries have delineated cuproptosis as a distinct form of cell death, uniquely precipitated by copper exposure (25). This process is markedly different from other established cell death mechanisms. Experimental validations have demonstrated that copper can provoke apoptosis through activation of aSMase, leading to subsequent ceramide release (26, 27). Despite these advancements, the connection between sphingolipid metabolism and cuproptosis remains nascent. However, it is understood that any dysregulation in sphingolipid metabolism might influence various cell death pathways. Since sphingolipids are crucial components of the cell membrane and affect multiple cellular signaling pathways, their metabolic changes may influence the cellular stress responses induced by copper. Understanding how sphingolipids regulate the pathways of copper-induced cell death will provide crucial insights into the molecular mechanisms of cuproptosis and the development of new anti-tumor strategies.

In elucidating the mechanistic progression of tumor diseases, multi-omics plays a pivotal role (28–30). By integrating multi-omic datasets with single-cell analysis methods, we explored the value of cuproptosis-related sphingolipid metabolism (cuproptosis-SPGs) in predicting outcomes and treatment efficacy in osteosarcoma patients, and revealed the impact of cuproptosis-SPGs on immune cell infiltration in osteosarcoma tissues. These studies provide a theoretical basis for future osteosarcoma research focusing on copper ion metabolism therapies, highlighting the interactions between copper ions and sphingolipid metabolism, and how they jointly regulate tumor cell behavior and immune system functionality. In-depth research in this field is expected to drive the development of a new generation of anti-tumor therapies.

## 2 Materials and methods

### 2.1 Data collection

To conduct our study on sphingolipid metabolism, a total of 160 genes were retrieved from the InnateDB (https://www.innatedb.com/), which are known to be implicated in this metabolic pathway. Additionally, transcriptomic datasets along with comprehensive clinical profiles for osteosarcoma patients were procured from the TARGET initiative (https://ocg.cancer.gov/programs/target). In total, transcriptomic profiles were obtained for 88 individuals diagnosed with osteosarcoma. However, complete clinical records were available for only 85 of these patients. Consequently, transcriptomic data from the remaining three patients were excluded from the study, thereby enabling subsequent analyses to proceed with the cohort of 85 osteosarcoma patients. The U-2 OS cell line, characterized by its epithelial morphology, originates from a sarcoma of moderate differentiation. This line exhibits adherent growth characteristics and displays significant chromosomal alterations. The osteosarcoma cell line U2OS (KCB200962YJ) was procured from Kmcellbank, serving as a crucial biological model for our analyses.

### 2.2 Establishment of risk scoring model

To evaluate the prognostic influence of distinct cuproptosis-SPGs, univariate Cox regression analyses were undertaken, linking their differential expression with survival durations (31). Genes demonstrating a significant association with survival metrics were identified as prospective elements for subsequent LASSO regression (32, 33). Selection of the optimal penalty parameter (λ) for the model was achieved through 10-fold cross-validation, utilizing a criterion of minimality. Computation of the final risk score was based on normalized expression values and coefficients of the selected variables. The formula is presented as follows: B4GALNT1*0.0314 + SGMS2*0.1847 + ABCA2*0.3275 - B3GALT4*0.0206 -ST3GAL2*0.1409 - NPC2*0.1068 - APOE*0.0882.

Following the development of the model, individuals in the TARGET-OS cohort were stratified into high-risk and low-risk categories, centered on the median score from the training set. Differences in survival rates between these categories were assessed using Kaplan-Meier survival plots and log-rank tests, facilitated by the “survival” and “survivalminer” software packages. Additionally, the model’s predictive accuracy was evaluated over 1, 3, and 5 years, using receiver operating characteristic (ROC) analysis (34, 35), implemented via an R package.

### 2.3 Decision curve analysis

To assess the clinical relevance of the prognostic framework, decision curve analysis (DCA) curves were generated using the “ggDCA” package. This methodology facilitates the identification of patient risk and quantifies the net benefit of the predictive model across varying threshold probabilities.

### 2.4 Functional enrichment analysis

For functional enrichment assessment, the “clusterProfiler” R package was employed to analyze functional enrichment based on differentially expressed genes (DEGs) between high-risk and low-risk groups. Additionally, single-sample Gene Set Enrichment Analysis (ssGSEA) was performed for each individual in both cohorts (36). Pathways that were significantly enriched were depicted through heatmaps illustrating the average ssGSEA scores.

### 2.5 Evaluation of immune landscape

The algorithms CIBERSORT and ESTIMATE were employed to determine the relative abundance and infiltration levels of immune cell subtypes, respectively (37). Spearman’s method was utilized to assess the correlation between the Riskscore and the degree of immune infiltration as per ESTIMATE. Additionally, the immune scores for TIL subgroups were derived using ssGSEA. Furthermore, ssGSEA in the GSVA packages was applied to quantify immune features within each sample.

### 2.6 Single-cell analysis of cuproptosis-SPGs expression in osteosarcoma tissues

For the processing of single-cell RNA sequencing data, we employed the R package Seurat (38, 39), version 4.1.0, to facilitate the log-normalization and standardization of single-cell transcriptomic profiles (40). Rigorous quality control measures were implemented, evaluating cells based on gene expression within the count matrix and the proportion of mitochondrial gene counts. Cells expressing more than 1500 genes were excluded, along with those exhibiting over 5% mitochondrial gene count. Subsequently, data normalization across the library size was conducted using the “normalize_total” function from the Scanpy toolkit. This normalization facilitated the generation of a logarithmically transformed data matrix, which was utilized in subsequent analytical processes. Ultimately, eight distinct cell types were annotated.

### 2.7 Drug sensitivity analysis and cell cytotoxicity detection

To investigate variations in drug responsiveness between low-risk and high-risk cohorts, we utilized the R package “pRRophetic” (41). Subsequently, differentially expressed genes (DEGs) distinguishing these groups were integrated into the Connectivity Map to pinpoint therapeutic agents that could potentially benefit patients at higher risk. Following this, the U2OS cell line was treated with roscovitine to assess the impact of cuproptosis-SPGs on drug sensitivity via cytotoxic assays.

### 2.8 Migration ability impacted by cuproptosis-SPGs

The impact of cuproptosis-SPGs on the migratory abilities of U2OS cells was evaluated utilizing both wound-healing and transwell migration assays (42). In the wound-healing assay, siRNA was employed to disrupt U2OS cells over a period of 72 hours. Subsequently, a deliberate “wound” was created within the cell monolayer, and cell migration rates were assessed after 48 hours. Additionally, siRNA-treated U2OS cells were placed in the upper chamber of a transwell setup. The lower chamber was supplied with 15% fetal bovine serum to serve as a chemoattractant for the U2OS cells. The number of cells present in the lower chamber after 36 hours was quantified to gauge cellular migration capabilities (43).

### 2.9 Statistical analysis

In this study, the analytical procedures were performed using RStudio, release 4.1.3. To assess overall survival differences, we utilized the log-rank test along with Kaplan-Meier survival analysis. For processing Western blot data, software such as ImageJ and GraphPad Prism were applied. Pearson’s method of correlation analysis was employed to execute all correlation assessments. Evaluation of disparities between the groups was conducted through the unpaired, two-tailed Student’s t-test, with a significance threshold set at P<0.05.

## 3 Results

### 3.1 Cuproptosis-SPGs for predicting osteosarcoma survivals

**Figure 1:**
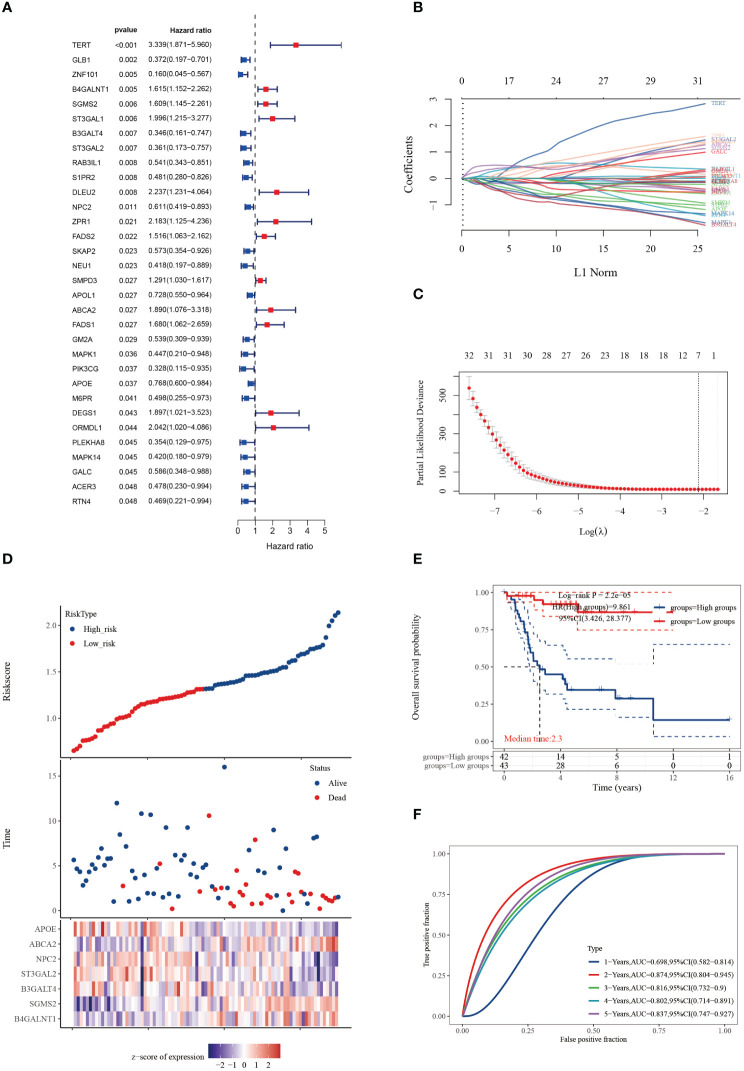
Cuproptosis-SPGs for predicting osteosarcoma survivals. **(A)** Differential gene analysis was employed to identify cuproptosis-SPGs correlated with osteosarcoma. **(B)** Coefficients for 32 cuproptosis-SPGs. **(C)** Employing the Lasso technique, a prognostic model based on 7 SRGs was formulated. **(D)** Expression levels of the foremost 7 cuproptosis-SPGs were graphically depicted to illustrate prognostic risk distribution. **(E)** KM plot was conducted to further explore the prognostic relevance of the 7 cuproptosis-SPGs across diverse osteosarcoma subtypes. **(F)** The prognostic model’s predictive efficacy was assessed via ROC analysis.

We conducted univariate Cox regression analysis on 158 cuproptosis-SPGs, identifying 32 of them as prognostically significant genes in osteosarcoma. Of these, 12 were associated with lower survival rates (**Figure 1A**). Subsequent to this identification, we employed LASSO regression modeling based on the same set of 32 cuproptosis-SPGs. This process culminated in the selection of seven core cuproptosis-SPGs for the construction of a prognostic model for osteosarcoma patients (**Figures 1B, C**). Multivariable Cox regression analysis elucidated the risk coefficients for these seven SPGs, categorizing patients into high and low-risk groups (**Figure 1D**). Kaplan-Meier survival plots revealed that patients in the high-risk category exhibited significantly poorer prognoses compared to those scored as low-risk (**Figure 1E**). The predictive capacity of the cuproptosis-SPGs model was evaluated using ROC curves. The model demonstrated impressive predictive accuracy with an AUC of 0.874 at two years and 0.837 at five years (**Figure 1F**).

### 3.2 Cuproptosis-SPGs model provides clinical benefits for osteosarcoma patients

**Figure 2:**
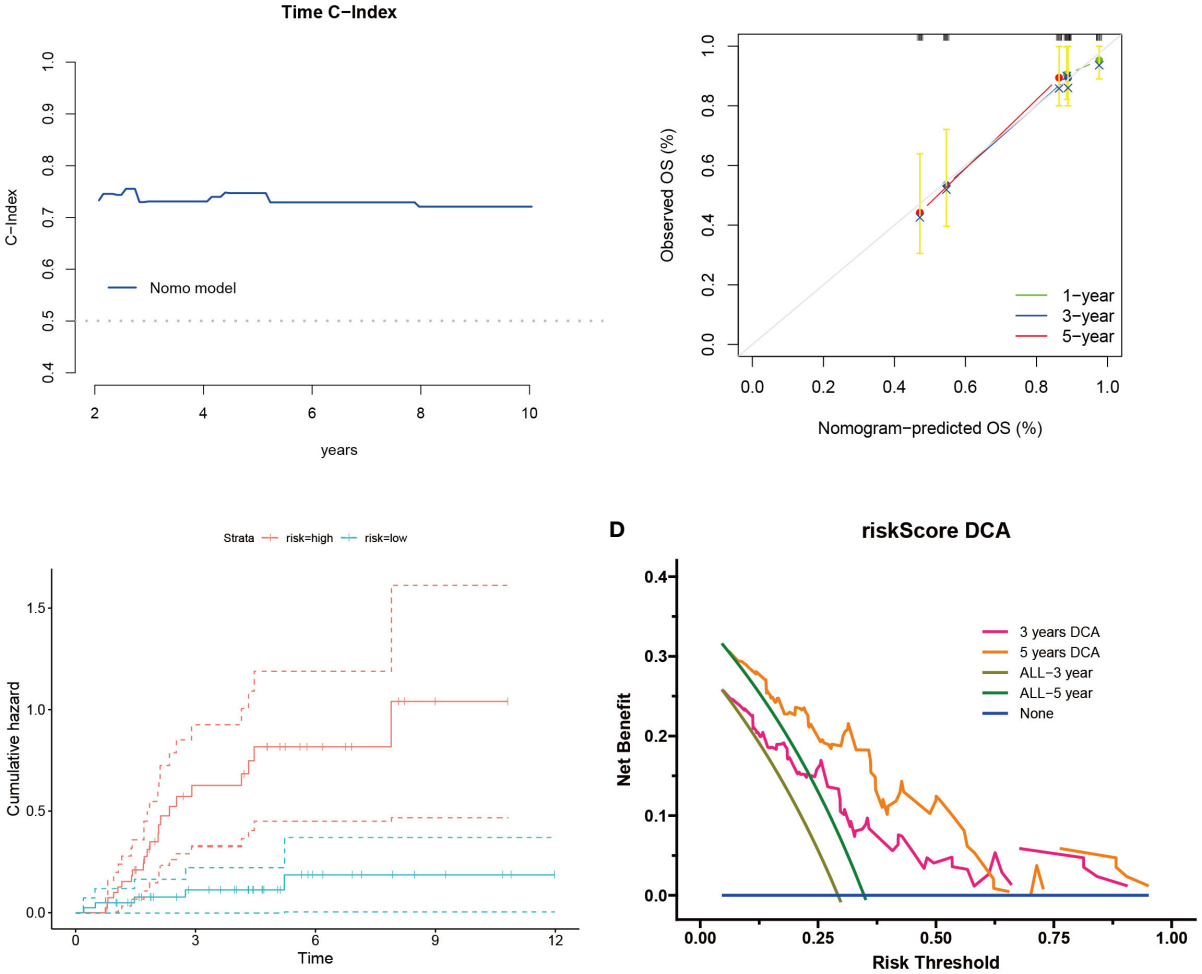
Cuproptosis-SPGs model provides clinical benefits for osteosarcoma patients. **(A)** Time C-Index plot showcases the model’s discriminative ability over time. **(B)** Calibration plot demonstrates the agreement between predicted and observed outcomes. **(C)** Cumulative risk curves illustrate the evolving risk estimates. **(D)** Decision curve analysis evaluates the net benefit of utilizing the model in clinical decision-making.

Following the construction of the prognostic model, we further assessed its overall accuracy and credibility using the C-index (**Figure 2A**). Calibration curves additionally demonstrated the predictive capacity of the model, showing good consistency with actual outcomes over 1, 3, and 5 years (**Figure 2B**). Moreover, osteosarcoma patients classified as high risk exhibited an increased cumulative hazard (**Figure 2C**), suggesting that Cuproptosis-SPGs may serve as a potential marker for the progression of osteosarcoma patients. Decision curve analysis indicated that the drug decisions based on nomograms provided greater benefits for osteosarcoma patients compared to other individual indicators (**Figure 2D**).

### 3.3 The classification of osteosarcoma groups based on Cuproptosis-SPGs reveals distinct immune networks

**Figure 3:**
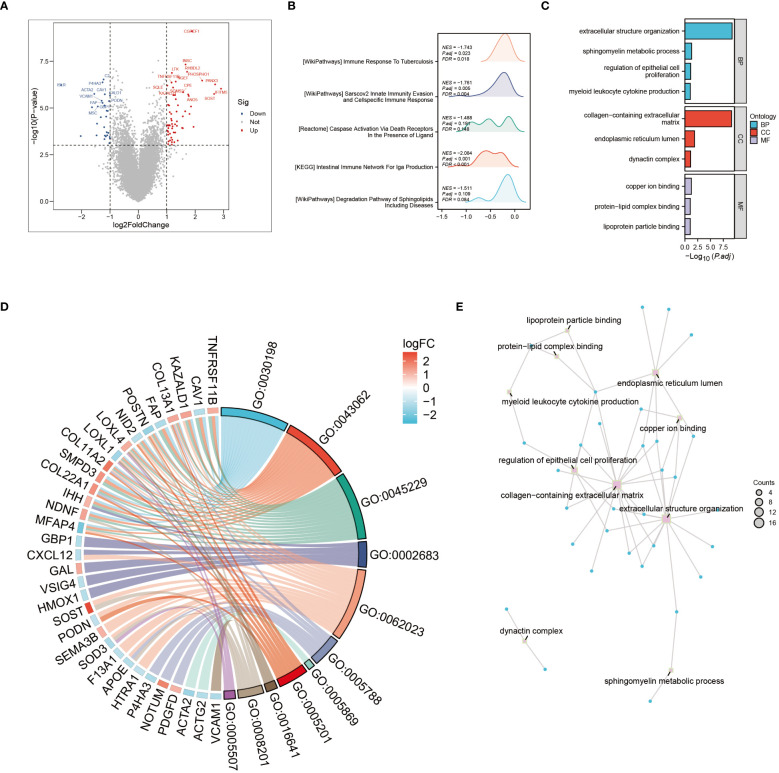
Go and KEGG enrichment analysis. **(A)** Volcano plot illustrating differential genes in high- and low-risk osteosarcoma patients. **(B)** Mountain plot depicting enrichment of differential genes in immune pathways. **(C)** Enrichment of differential genes in Gene Ontology (GO) terms related to Biological Processes (BP), Cellular Components (CC), and Molecular Functions (MF). **(D)** Pathways enriched in GO analysis and corresponding differential genes. **(E)** Interconnections between enriched pathways.

The tumor microenvironment plays a pivotal role in the development and progression of cancer. In this study, patients with osteosarcoma were stratified into high-risk and low-risk categories utilizing the Cuproptosis-SPGs model. Differential gene expression analysis was conducted for both groups, followed by functional enrichment studies. This approach identified 79 differentially expressed genes (DEGs), the majority of which were upregulated (**Figure 3A**). Functional enrichment of these DEGs highlighted extensive involvement in immune response processes and sphingolipid metabolism between the high and low-risk groups (**Figures 3B, C**). Among these, four Gene Ontology (GO) processes showed significant alterations (**Figure 3D**). The copper ion binding process and sphingomyelin metabolic process are linked through the extracellular structure organization, suggesting that the extracellular structure may act as a bridge in the sphingolipid-cuproptosis network (**Figure 3E**).

### 3.4 Interactions between cuproptosis and sphingolipid metabolic genes

**Figure 4:**
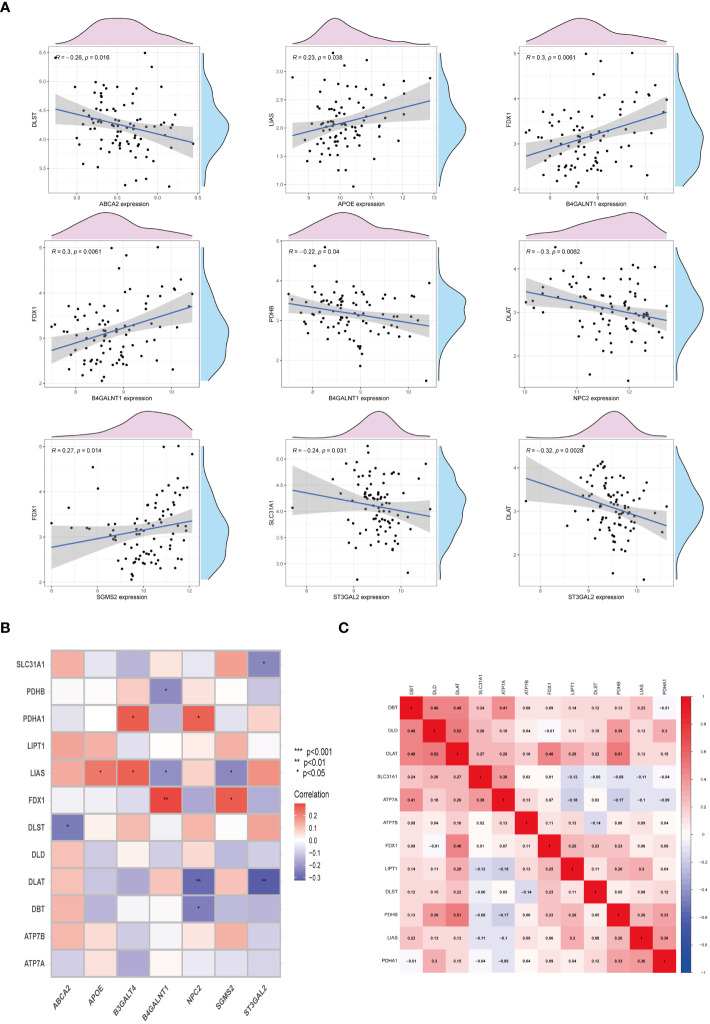
Interactions between cuproptosis and sphingolipid metabolic genes. **(A, B)** Relationship of cuproptosis-SPGs and cuproptosis genes. **(C)** Heatmap exhibits the association among cuproptosis-SPGs. (*P < 0.05, **P < 0.01).

To elucidate the interplay between cuproptosis metabolism and sphingolipid metabolism, correlations between genes involved in cuproptosis and sphingolipid pathways were investigated. As depicted in **Figure 4A**, a notable, albeit negative, correlation persists between these gene sets. Noteworthy, the correlation between FDX1 and B4GALNT1 was found to be the most significant (P < 0.01) (**Figure 4B**). Additionally, the heatmap in **Figure 4C** illustrates the correlation levels among cuproptosis genes, with DAL and DLD exhibiting the highest correlation coefficient (R = 0.52).

### 3.5 Single-cell analysis exhibits Cuproptosis-SPGs expression levels in tumor microenvironment

**Figure 5:**
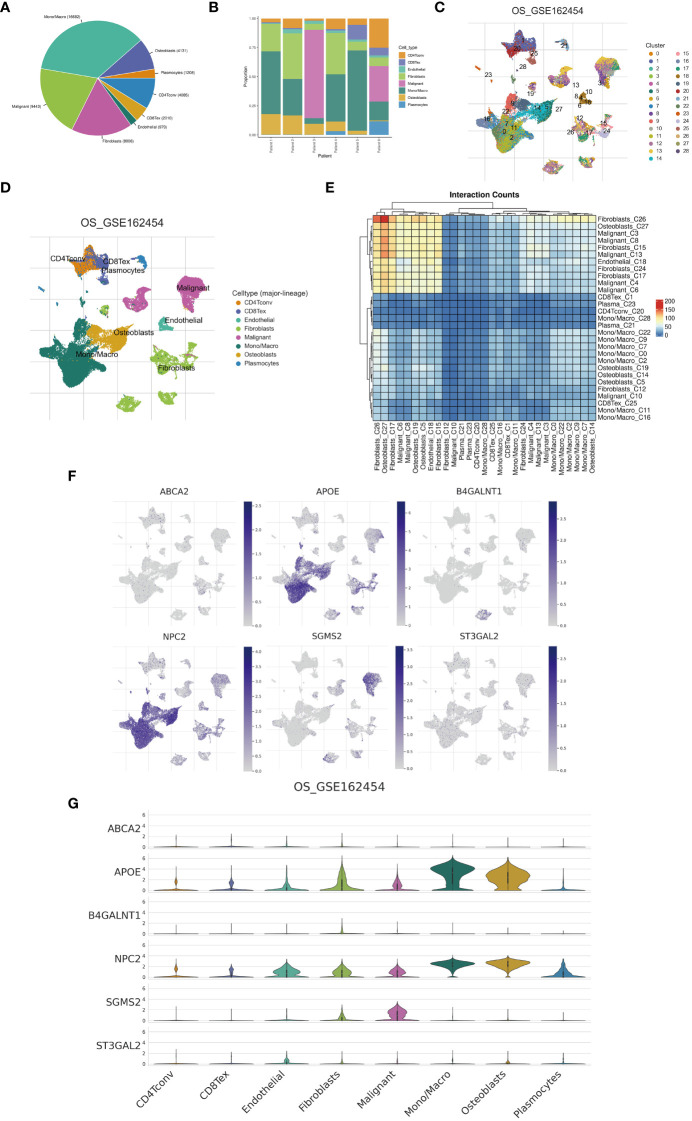
Single-cell analysis. **(A)** A pie chart illustrates the proportional representation of all cellular subpopulations. **(B)** Bar graphs depict the distribution of cellular subpopulations for each osteosarcoma patient. **(C, D)** UMAP visualization demonstrates the clustering of cellular subpopulations. **(E)** A heatmap displays the interaction counts among cellular subpopulations. **(F, G)** Distribution patterns of cuproptosis-SPGs between different cells are depicted.

**Figure 6:**
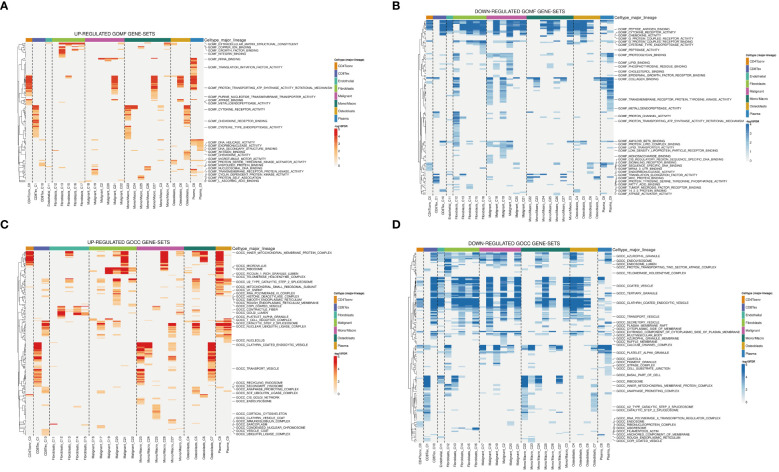
Analysis of GO terms in osteosarcoma patients. **(A, B)** Upregulated and downregulated gene sets in GOMF. **(C, D)** Upregulated and downregulated gene sets in GOCC.

**Figure 7:**
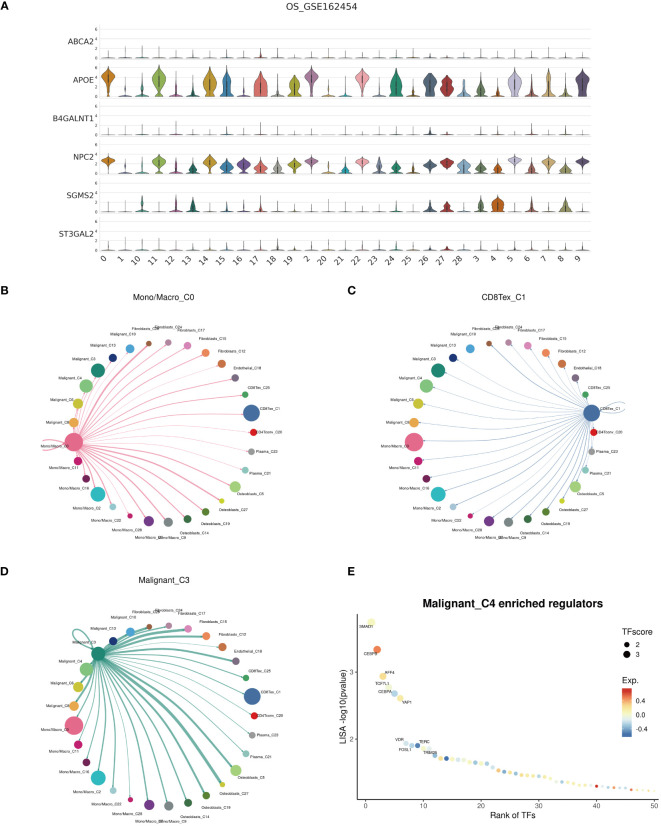
Cell communication. **(A)** Expression profiles of cuproptosis-SPGs within cellular subgroups clusters. **(B–D)** Interactions among Mono/Macro, T cells, and malignant cells within the osteosarcoma cellular subgroups. **(E)** Transcription factor regulation of osteosarcoma cells.

Examination at the level of individual cells has unveiled a significant presence of T cell and monocyte/macrophage subsets within the immune context of osteosarcoma (**Figures 5A, B**). By employing UMAP analysis, we identified 28 unique clusters across eight cellular subtypes, revealing diverse subpopulations of cells (**Figures 5C, D**). Employing heatmap visualization methods, distinctive patterns of interaction between osteoblasts and fibroblasts were emphasized (**Figure 5E**). To delve into the expression patterns of SRGs within the tumor microenvironment in osteosarcoma, we explored the expression levels of the top six ranked SRGs in each cell subtype (**Figures 5F, G**). A pivotal finding was the notable decrease in peptide antigen binding pathways (**Figures 6A, C**), coupled with a notable increase in ATP synthase activity in T cells (**Figures 6B, D**). Analysis of SRGs showed a consistent rise in APOE expression among different cell types (**Figure 7A**). Given the variety of T cell subsets discovered in osteosarcoma, additional studies were initiated to explore the interactions between these lymphocytes and the tumor cells (**Figures 7B–D**). Investigative efforts into the transcription factors in tumor cells highlighted potential pathways influencing differential expression (**Figure 7E**).

### 3.6 Alterations in CD8 T Cell infiltration mediated by Cuproptosis-SPGs

**Figure 8:**
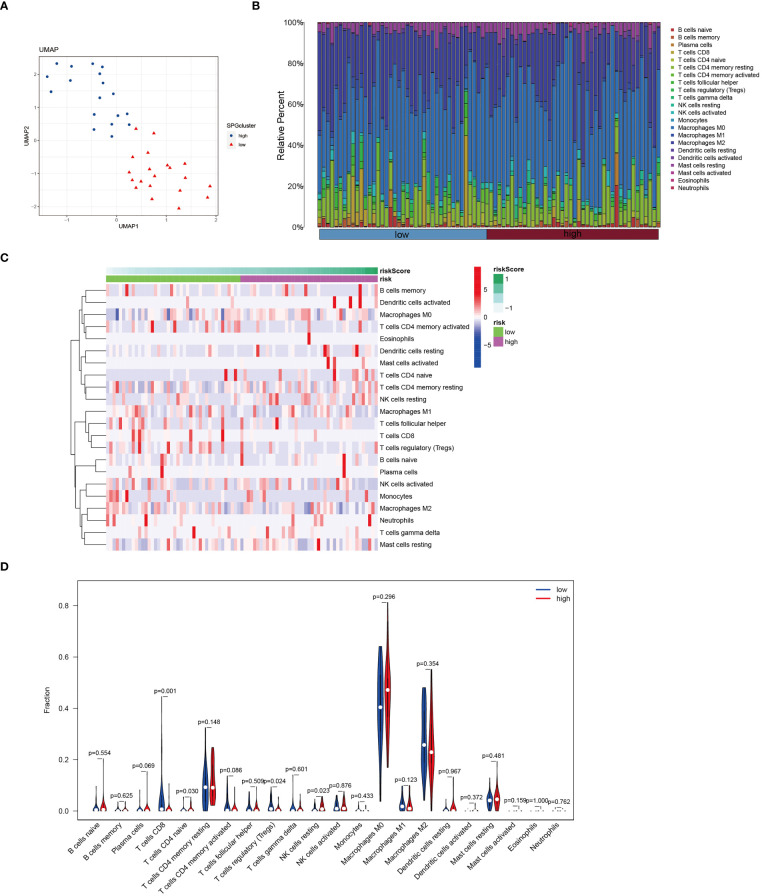
Immune features of cuproptosis-SPGs classified osteosarcoma subtypes. **(A)** UMAP visualization showcases the expression levels of cuproptosis-SPGs within immune cells. **(B)** CIBERSORT reveals the proportions of immune cell components in osteosarcoma patients. **(C)** The level of immune cell infiltration for each osteosarcoma patient is illustrated. **(D)** Variations in immune cell infiltration between high-risk and low-risk osteosarcoma patients are delineated.

**Figure 9:**
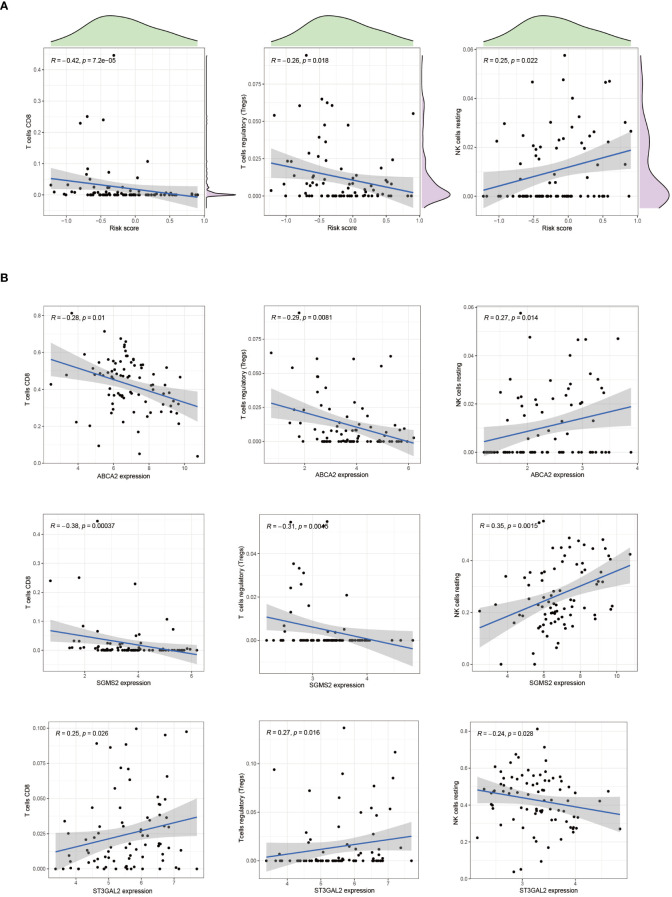
The association between immune cells and riskscore. **(A)** Correlation exists between CD8 T cells, Treg cells, NK cells, and riskscore. **(B)** The correlation between CD8 T cells, Treg cells, NK cells, and cuproptosis-SPGs.

In patients with osteosarcoma, there exists a stark contrast in the levels of Cuproptosis-SPGs between those at high risk and those at low risk (**Figure 8A**). An estimation of cellular proportions indicates a diminished presence of CD8+ T cells in the high-risk group (**Figure 8B**). Analyses of the tumor immune microenvironment (TME) reveal significant variances not only in CD8 T cells but also in NK cells and Tregs across different risk categories of osteosarcoma patients (**Figures 8C, D**). These differences were corroborated by an analysis linking risk scores with immune cell correlations (**Figure 9A**). Given that risk scores are derived from Cuproptosis-SPGs, further investigation into the association between Cuproptosis-SPGs and the infiltration levels of CD8 T cells, NK cells, and Tregs was conducted. The results demonstrate a significant negative correlation between ABCA2 and the infiltration of CD8 T cells (**Figure 9B**), suggesting that ABCA2 may be a critical Cuproptosis-SPG gene influencing anti-tumor immunity.

### 3.7 Drug response differences and validations

**Figure 10:**
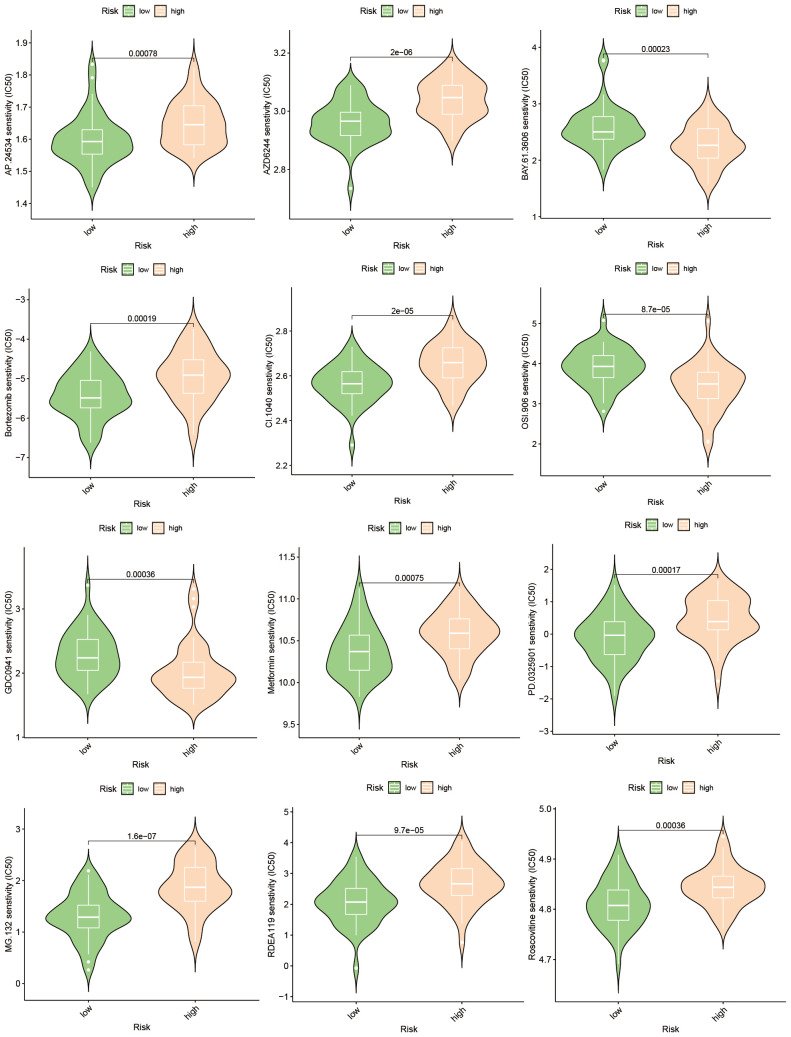
Drug sensitivity prediction.

**Figure 11:**
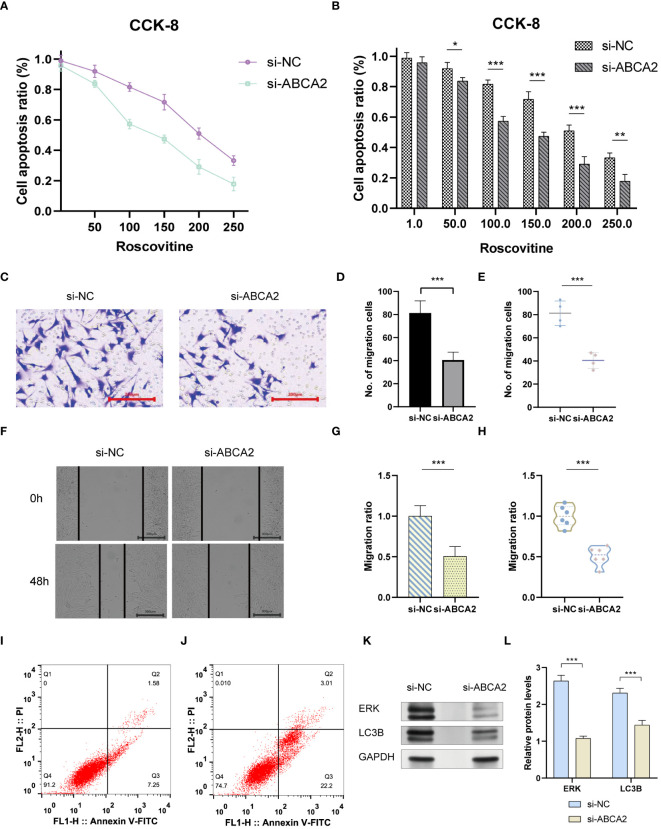
The impact of ABCA2 on tumor migration ability. **(A)** CCK-8 cell viability examination. **(B)** Bar graph showing the synergistic effect of ABCA2 inhibition at different concentrations of Roscovitine. **(C)** Transwell assay demonstrating osteosarcoma cells crossing through chambers after interference with ABCA2. **(D, E)** Bar graph and scatter plot illustrating the influence of ABCA2 on osteosarcoma cell migration capability. **(F)** Wound-healing assay showing migrated osteosarcoma cells after interference with ABCA2. **(G, H)** Bar graph and scatter plot demonstrating the inhibition of osteosarcoma cell migration capability by ABCA2. **(I, J)** Flow cytometry tests. **(K, L)** Western blot shows the influence of inhibition of ABCA2. (*P < 0.05, **P< 0.01, ***P< 0.001).

The emergence of chemoresistance presents a significant challenge in the treatment of tumors. Our predictive analyses suggest that patients with high-risk osteosarcoma exhibit enhanced sensitivity to roscovitine, metformin, and bortezomib compared to those with low-risk osteosarcoma (**Figure 10**). Given the substantial weight of ABCA2 in the risk assessment, further investigations were conducted to explore its role in mediating drug sensitivity in osteosarcoma. After transfection with siRNA, osteosarcoma U2OS cells were exposed to varying concentrations of roscovitine for 48 hours. Cytotoxicity assays revealed that, relative to controls, cells with ABCA2 knockdown demonstrated reduced cytotoxic response to roscovitine (**Figure 11A**), with more pronounced differences at concentrations between 100–200 µM (**Figure 11B**). These findings align with the predictions from the drug sensitivity model, indicating that osteosarcoma patients with elevated ABCA2 expression are more susceptible to roscovitine. Future research should elucidate the mechanisms by which ABCA2 influences drug sensitivity through its interactions with pathways such as cuproptosis and sphingolipid metabolism.

### 3.8 Cuproptosis-SPG regulates osteosarcoma cells migration ability

Following the validation of the drug sensitivity model using siRNA, the precision of the Cuproptosis-SPGs prognostic model was assessed. It was observed that 48 hours post siRNA treatment, the number of U2OS cells traversing the compartments was diminished (**Figure 11C**) and exhibited statistically significant differences (**Figures 11D, E**). Consistent with the transwell assay results, wound healing experiments indicated that inhibition of ABCA2 curtailed the migration distance of U2OS cells (**Figure 11F**), thus underscoring the role of ABCA2 in facilitating U2OS cell migration (**Figures 11G, H**).

### 3.9 Cuproptosis-SPG regulation of autophagy and MAPK proteins affects apoptosis in U2OS cells

Tumor cells evade apoptosis by remodeling their metabolism. We assessed the impact of the Cuproptosis-SPG gene ABCA2 on apoptosis in osteosarcoma cells using flow cytometry. It was observed that U2OS cells with disrupted ABCA2 were more susceptible to apoptosis, exhibiting an increased apoptotic rate of 16.39% compared to the control group (**Figures 11I, J**). Interestingly, after ABCA2 knockdown, autophagy levels in U2OS cells were also suppressed, and this was associated with hindered expression of ERK proteins (**Figures 11K, L**). These findings suggest that Cuproptosis-SPG may regulate the apoptotic process in osteosarcoma cells through both the autophagy and the MAPK pathways.

### 3.10 Cuproptosis-SPG expression patterns in pan-cancer

We further tried to explore the potential utility of Cuproptosis-SPG across additional tumor contexts. Our investigations incorporated an expression analysis of ABCA2 in normal tissues (**Supplementary Figure S1A**). The findings indicate a predominant expression of ABCA2 in immune cells, such as Gdh T cells (**Supplementary Figure S1B**). Notably, within a pan-cancer analysis, ABCA2 exhibited a significantly elevated expression in CHOL compared to normal tissues (**Supplementary Figure S1C**). Despite this, the correlation between ABCA2 expression levels and prognosis in CHOL was minimal (**Supplementary Figure S1D**).

## 4 Discussion

Osteosarcoma is characterized by complex epigenetic features and a high propensity for metastasis, frequently occurring in adolescents (44). Currently, chemotherapy and surgical interventions are effective predominantly in early-stage osteosarcoma patients (45). Those diagnosed with metastatic or recurrent osteosarcoma continue to face grim outcomes, with less than 30% achieving long-term survival (46). Clinical advancements for patients with advanced-stage osteosarcoma remain limited. Immunotherapy, as an emerging therapeutic approach, has yet to be extensively explored in osteosarcoma (47, 48).

In this study, we focused on the prognostic capabilities of copper metabolism and sphingolipid metabolism networks in osteosarcoma patients, elucidating the potential mechanisms through which cuproptosis-related sphingolipid genes (cuproptosis-SPGs) impact patient outcomes. We initially identified seven cuproptosis-SPGs and developed a prognostic model demonstrating robust predictive performance. Indeed, effective prediction models are crucial for the treatment of osteosarcoma patients, given the significant variability in pathological patterns among patients, which markedly influences treatment outcomes (49–51). Previous studies have shown that single genes or proteins can exhibit strong prognostic abilities (52–54). Metabolic alterations are key factors in cancer development and progression (55, 56), with sphingolipid metabolism playing a broad role in disease onset and serving a critical function in cancer progression and prognosis. It has been demonstrated that sphingolipid metabolism can modulate iron metabolism (57), such as ferroptosis, thereby inducing autophagy-related changes that lead to drug resistance (58). Additionally, sphingolipid metabolism regulates T lymphocyte calcium channels, contributing to tumor immune evasion (13, 59). Studies have explained the network between copper and aSMase, indicating the potential regulation mechanism in diseases’ development. However, no studies have yet disclosed the link between sphingolipid and copper metabolism in osteosarcoma. In our research, we identified seven cuproptosis-related sphingolipid metabolism genes (cuproptosis-SPGs) and clarified the connection between cuproptosis and sphingolipids (**Figure 4**). Extracellular structure organization may be a crucial hub in the cuproptosis-SPGs process (**Figure 3E**). The extracellular matrix (ECM) and other components provide structural and biochemical support to surrounding cells, facilitate intercellular communication, and influence cellular functions such as differentiation, migration, and adhesion. Sphingolipids are involved in the formation of focal adhesions (FAs), affecting the migratory capacity of cancer cells (60, 61). Our study found that knockdown of the cuproptosis-SPG gene ABCA2 significantly inhibited the migration ability of osteosarcoma cells (**Figures 11C, F**), suggesting that the sphingolipid-cuproptosis metabolic network jointly influences the formation of focal adhesions, thereby regulating cancer cell migration.

Drug resistance remains one of the greatest obstacles to successful cancer therapy (62, 63). Due to tumor heterogeneity, the molecular expression patterns vary among cancer patients (64–66), leading to significant differences in responses to anti-cancer treatments (67, 68). Based on cuproptosis-SPGs, we stratified osteosarcoma patients into high-risk and low-risk groups. The study demonstrated that the sphingolipid-cuproptosis metabolic network affects osteosarcoma patients’ drug sensitivity (**Figure 10**). Research by Vu NT et al. found that sphingolipids can regulate mitochondrial functions to drive ferroptosis in tumors, thereby altering tumor cell sensitivity to cisplatin (58). Our research is the first to reveal the significant role of the sphingolipid-cuproptosis metabolic network in osteosarcoma drug sensitivity and preliminarily identified autophagy and MAPK as key downstream regulatory pathways (**Figure 11K**).

Osteosarcoma patients exhibit unique immune infiltration characteristics associated with poor prognosis (69). Stratification of cancer patients based on multi-omics data facilitates the guidance of precision treatment strategies (70–72). Our study, using seven core cuproptosis-SPGs, developed a prognostic model that demonstrated excellent predictive performance (**Figure 1F**), offering clinical benefits to stratified osteosarcoma patients. Interestingly, different stratifications of osteosarcoma patients showed distinct immune infiltration. As delineated by the single-cell analysis, monocytes/macrophages represent the predominant cell type within the osteosarcoma tissue matrix, followed by CD8+ T cells (**Figure 5A**). These observations suggest that cuproptosis-SPGs may predominantly influence the infiltration levels of these two cell types. Subsequent multi-omic analyses revealed that within the macrophage population, the M0, M1, and M2 phenotypes indeed constituted the majority (**Figure 8B**). However, no significant differences were observed in the macrophage infiltration levels between the high-risk and low-risk groups, indicating that macrophage seems to influence limited in the progression of osteosarcoma. Notably, CD8 T cells, crucial for effective recognition and killing of tumor cells during the immune response (73, 74), were observed to have lower infiltration levels in high-risk osteosarcoma patients compared to those at low risk (**Figure 8D**). This suggests that the sphingolipid-cuproptosis metabolic network participates in the recruitment of CD8 T cells. Therefore, targeting cuproptosis-SPGs may enhance the efficacy of immunotherapies such as CAR-T in combating osteosarcoma.

Although our study has revealed the impact of cuproptosis-SPGs on the immune microenvironment and drug sensitivity in osteosarcoma, and has preliminarily identified potential regulatory mechanisms, there are several limitations to be acknowledged. Firstly, more osteosarcoma samples need to be collected to adjust the reliability and applicability of the model. Secondly, despite single-cell analysis revealing the immune infiltration landscape, these conclusions still require further validation in *in vivo* experiments. Similarly, experiments on drug sensitivity also need further validation in *in vivo* experiments. In future studies, we will delve deeper into *in vivo* mechanistic research to elucidate the significant role of cuproptosis-SPGs in the progression of osteosarcoma.

## 5 Conclusion

In summary, by integrating multi-omics datasets and single-cell analysis methods, our study explored the interplay between cuproptosis and sphingolipid metabolism and identified seven cuproptosis-related sphingolipid metabolism genes (cuproptosis-SPGs), used to construct a prognostic model for osteosarcoma patients. Our results delineate the value of this model in predicting patient outcomes and treatment efficacy, elucidate the regulation of immune cell infiltration by cuproptosis-SPGs, and validate these effects through cellular experiments. This study provides a theoretical foundation for future osteosarcoma studies focusing on copper ion metabolism therapies, highlighting the interactions.

## Data availability statement

The original contributions presented in the study are included in the article/**Supplementary Material**. Further inquiries can be directed to the corresponding authors.

## Ethics statement

Ethical approval was not required for the studies on humans in accordance with the local legislation and institutional requirements because only commercially available established cell lines were used.

## Author contributions

QL: Data curation, Formal analysis, Investigation, Software, Visualization, Writing – original draft. JF: Data curation, Formal analysis, Visualization, Writing – original draft. KL: Investigation, Resources, Writing – original draft. PL: Conceptualization, Funding acquisition, Supervision, Validation, Writing – review & editing. XW: Conceptualization, Investigation, Methodology, Project administration, Supervision, Validation, Writing – review & editing.
