# Supplementary figures and images for: Multi-omic validation of the cuproptosis-sphingolipid metabolism network: modulating the immune landscape in osteosarcoma
Source: Front Immunol. 2024 Jun 25;15:1424806. doi: 10.3389/fimmu.2024.1424806 (PMC11231095; doi:10.3389/fimmu.2024.1424806)

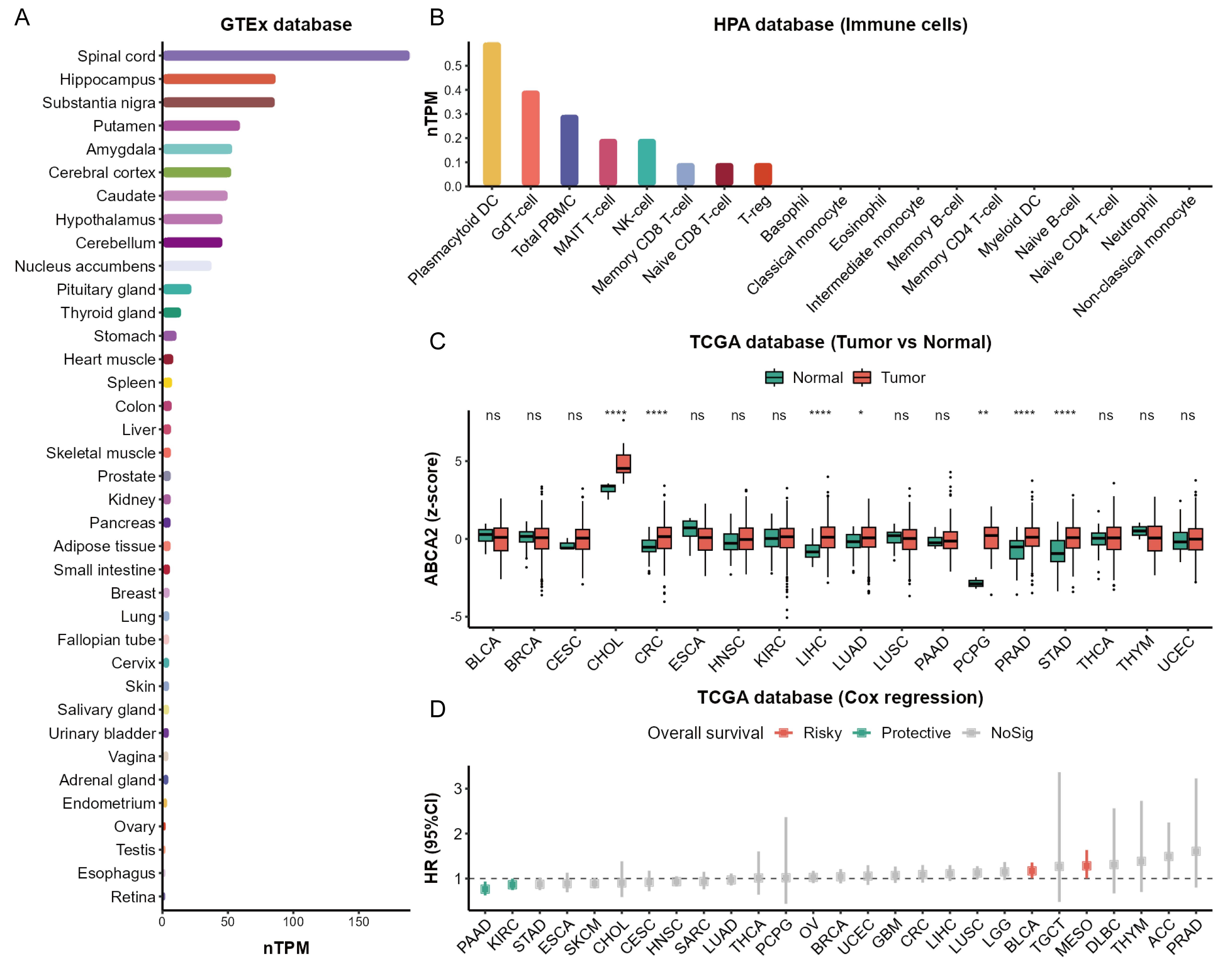

Supplement: Supplementary file 1 [file Image_1.tif]
